# Cellular Reactive Oxygen Species Inhibit MPYS Induction of IFNβ

**DOI:** 10.1371/journal.pone.0015142

**Published:** 2010-12-10

**Authors:** Lei Jin, Laurel L. Lenz, John C. Cambier

**Affiliations:** Integrated Department of Immunology, University of Colorado Denver School of Medicine and National Jewish Health, Denver, Colorado, United States of America; Charité-University Medicine Berlin, Germany

## Abstract

Many inflammatory diseases, as well as infections, are accompanied by elevation in cellular levels of Reactive Oxygen Species (ROS). Here we report that MPYS, a.k.a. STING, which was recently shown to mediate activation of IFNβ expression during infection, is a ROS sensor. ROS induce intermolecular disulfide bonds formation in MPYS homodimer and inhibit MPYS IFNβ stimulatory activity. Cys-64, -148, -292, -309 and the potential C_88_xxC_91_ redox motif in MPYS are indispensable for IFNβ stimulation and IRF3 activation. Thus, our results identify a novel mechanism for ROS regulation of IFNβ stimulation.

## Introduction

ROS, including the superoxide anion, hydrogen peroxide and hydroxyl radicals, are generated from the incomplete reduction of oxygen [1]. Most intracellular ROS (90%) are generated in mitochondria due to electron leakage along the respiratory chain [2]. ROS are also generated in the endoplasmic reticulum (ER) during the unfolded protein response (UPR) [3]. Bacterial infections induce transient production of large amounts of ROS, a process called the oxidative burst, on the membrane of endosomes within phagocytes such as neutrophils and macrophages [4].

ROS are a double-edged sword. Potent ROS mediated oxidative stress causes irreversible cell damage and eventually cell death [5]. More modest elevation of ROS activates “redoxin signaling” which uses cysteine residues as redox sensors to mediate inflammatory responses [1], [5]. Cys can be reversibly oxidized to sulfenic acids, S-glutathionylated or S-nitrosylated cysteines, or disulfide bonds [1]. S-glutathionylations of IRF3 and Cys-179 of the IKK-β subunit inhibit their activation [6], [7]. Oxidation of catalytic Cys in caspases and protein tyrosine phosphatases (PTP) inhibits their activation [8], [9]. Thus, ROS-mediated post-translational modifications on Cys regulate the biological activities of many proteins. High cellular ROS levels have been linked to ageing, human cancers, inflammatory, lung and cardiovascular diseases [5], [10]. Antioxidants show protective effects for certain cancers and cardiovascular diseases [11].

MPYS is a four-transmembrane protein which was originally identified as a growth inhibitor that mediates anti-MHC II mAb induction of cell death in B lymphoma cells [12]. Later, it was found MPYS is a potent IFNβ stimulator that is essential for innate immune responses to RNA and DNA viruses [13]. MPYS contains a potential redox-active Cys_88_-L-G-Cys_91_ (C_88_xxC_91_) motif at the N-terminus of the second TM (Figure S3). The CxxC motifs are often found in the oxidoreductases and are essential for their catalysis of redox reactions [14]. Here we report that MPYS is a ROS sensor. Sustained cellular ROS cause MPYS oxidation and loss of its ability to activate IFNβ expression.

## Results

### ROS inhibit MPYS induction of IFNβ expression

**Figure 1:**
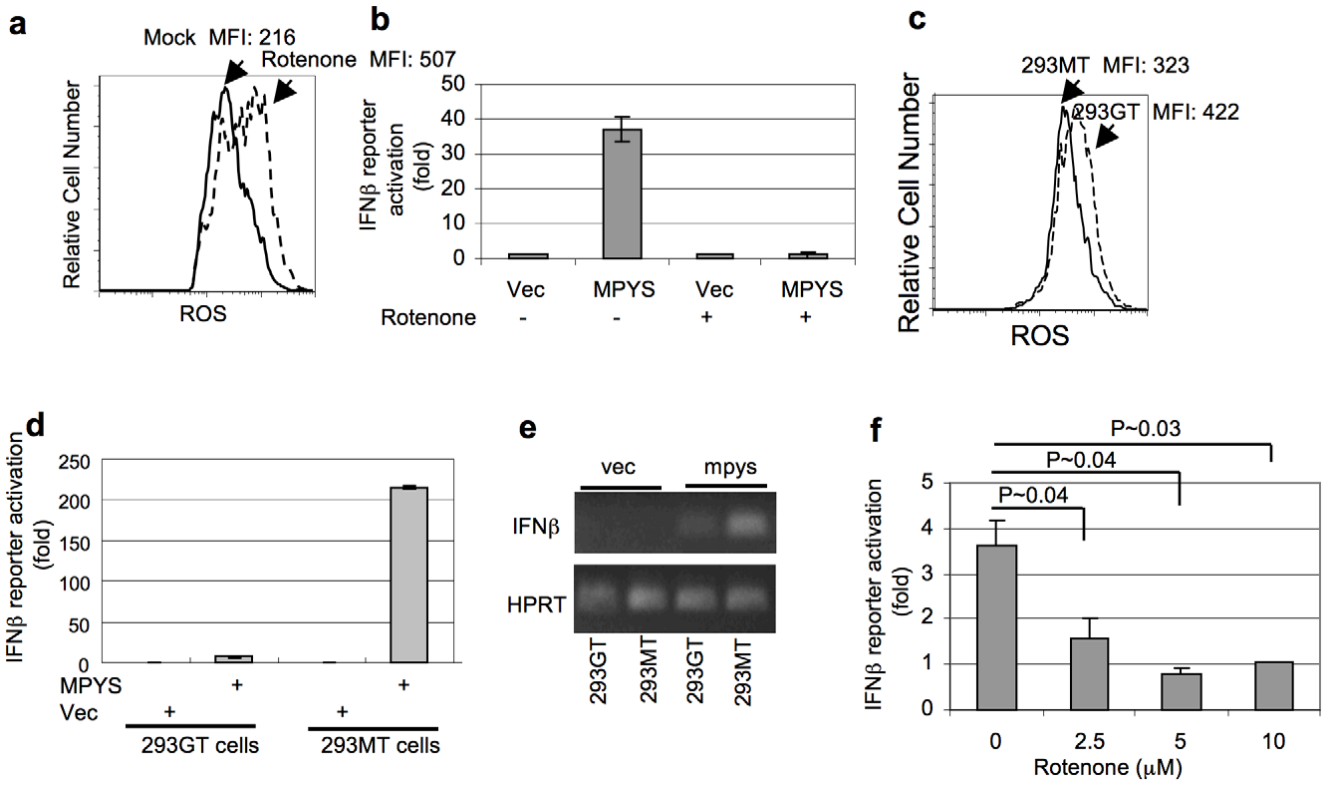
ROS inhibits MPYS mediated IFNβ production. **a**. 293T cells were first transfected with HA-MPYS for 8 hrs. Rotenone (10 µM) was then added in the culture for another 36 hrs. Intracellular ROS was measured by H_2_DCFDA (5 µM). **b**. 293T cells were transfected with indicated plasmids and luciferase reporters for 8 hrs. Rotenone (10 µM) was then added into the culture for another 36 hrs. The luciferase activities were measured afterwards. Error bars represent SD of a duplicate. **c**. 293GT or 293MT cells were stained with H_2_DCFDA (5 µM). **d**. 293MT or 293GT cells were transfected with indicated plasmids along with IFNβ luciferase reporter construct. Luciferase activity was measured as before. Error bars represent SD of a duplicate. **e**. RT-PCR was performed in cells transfected with indicated plasmid after 24 hrs as described in *Materials and Methods*. **f**. RAW264.7-IFNβ-Luc cells were first treated with rotenone for 16 hrs. *Listeria monocytogenes* infection and luciferase activity measurement were performed as in *Materials and Methods*. P value was calculated by student T-test (one-tailed).

MPYS was initially located in mitochondria [12]. Mitochondria is the major source for intracellular ROS production due to electron leakage along the respiratory chain [2]. We asked if the function of MPYS can be regulated by ROS. Rotenone is a specific inhibitor of mitochondrial electron transport chain complex I [15]. 293T cells treated with rotenone exhibited increased cellular ROS level (Figure 1a). MPYS is an IFNβ stimulator. Overexpressing MPYS in 293T cells activates IFNβ promoter [13]. However, we found that pre-treatment of 293T cells with rotenone completely abolishes MPYS induction of IFNβ (Figure 1b).

To test the hypothesis that ROS interfere with MPYS function, we expressed MPYS in two different lines: 293GT and 293MT. The 293GT line has constitutively higher endogenous ROS than the 293MT line (Figure 1c). We found that in 293GT cells, MPYS lost its ability to activate IFNβ (Figure 1d, 1e).

We next determined whether ROS compromise MPYS ability to transduce signals in response to a physiologic stimulus. MPYS is required for *Listeria monocytogen*es induced IFNβ response (Figure S1c) [13]. We found that pre-treatment of rotenone significantly inhibited *Listeria monocytogenes* induction of IFNβ production in RAW264.7 macrophage cells (Figure 1f). As a control, Rotenone pre-treatment did not inhibit Poly (I:C) induced IFNβ response (Figure S1d). We conclude that sustained cellular ROS inhibit MPYS induction of IFNβ.

### ROS induce MPYS oxidation

**Figure 2:**
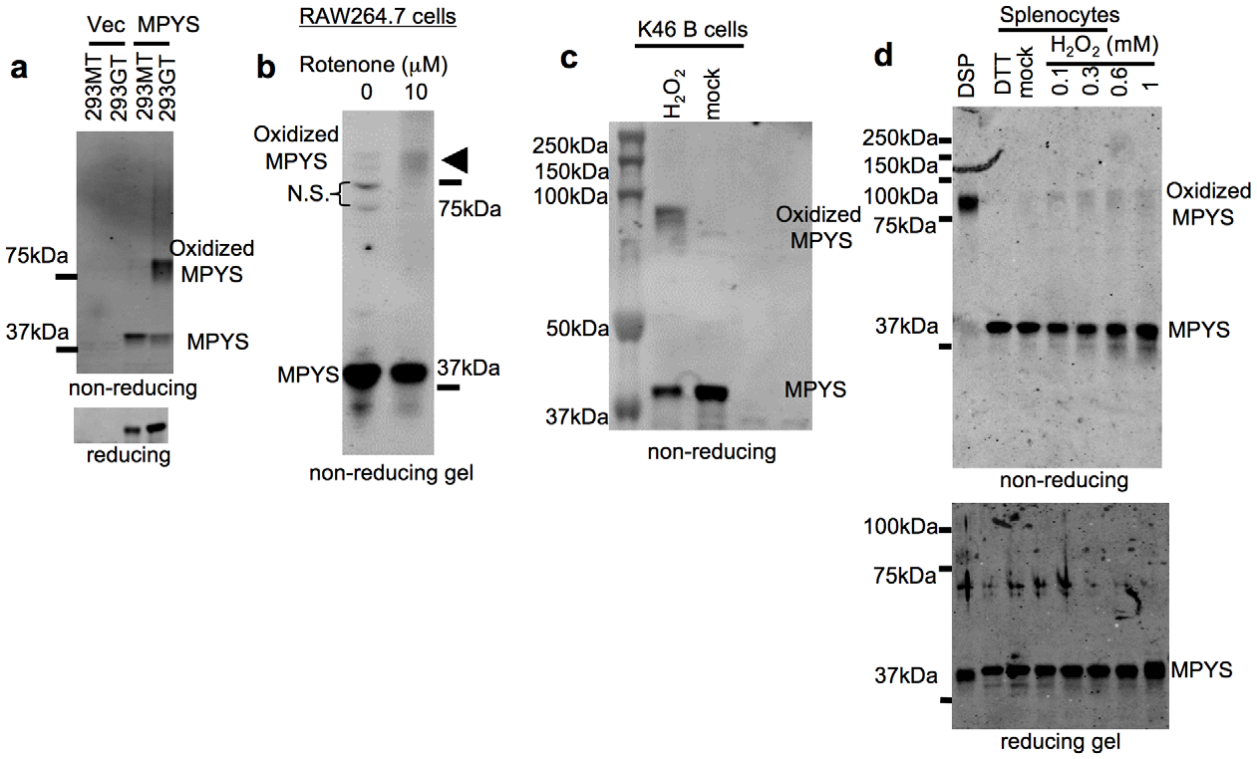
ROS induce MPYS oxidation. **a**. 293GT or 293MT cells expressing indicated plasmids were lysed in the RIPA buffer, fractionated on a non-reducing gel and probed with anti-MPYS Ab. **b**. RAW264.7-IFNβ-Luc cells were treated with rotenone as in **Fig. 1f**. Oxidized MPYS was examined as in (**a**). **c**. K46 B lymphoma cells were treated with H_2_O_2_ (1 mM) at 37°C in PBS for 20 min then lysed in RIPA buffer. Oxidized MPYS was examined as in (**a**). **d**. Murine splenocytes were suspended in PBS (10^6^ cells/ml) and treated with DTT (1 mM), no stimulus (mock) or the indicated amount of H_2_O_2_ at 37°C for 30 min. DSP (400 µM) crosslinking was performed at room temperature for 20 min. Cells were lysed in the RIPA buffer (50 mM pH 7.4 Tris-Cl, 1% NP40, 0.25% sodium deoxycholate, 150 mm sodium chloride and 0.1% SDS). Oxidized MPYS was examined as in (**a**).

We suspected that MPYS maybe a direct target of cellular ROS because on non-reducing gel, MPYS runs mainly as a ∼80 kDa SDS-resistant form in the 293GT cells, which has high endogenous ROS level (Figure 2a, lane 4), while in 293MT cells, MPYS runs as a ∼40 kDa protein (Figure 2a, lane 3). Also, treatment of RAW264.7 cells with rotenone induces the formation of that ∼80 kDa MPYS complex (Figure 2b). This ∼80 kDa SDS resistant MPYS (referred as oxidized MPYS) was also formed in K46 mouse B lymphoma cells (Figure 2c) upon H_2_O_2_ treatment. ER stress inducer, Brefeldin A, and serum starvation, also induce oxidized MPYS formation (Figure S2a, S2b).

### Endogenous MPYS occurs predominantly as a non-disulfide-bonded homodimer

We showed previously that MPYS is predominantly dimeric in mouse B cells [12]. Using the reducible chemical crosslinker Dithiobis [succinimidyl propionate] (DSP), we found on non-reducing SDS-PAGE, DSP treated MPYS ran as a single band of ∼80 kDa, the predicted size of a MPYS homodimer (Figure 2d, lane 1) while non-DSP treated mock (or DTT treated) MPYS ran at the monomer size (∼40 kDa) (Figure 2d, lane 3, 2). We further found that Flag-tagged MPYS associates with differently tagged, i.e. HA-MPYS, when the two are co-expressed (Figure S2c). Thus, the ∼80 kDa band seen upon non-reducing SDS-PAGE gel analysis of DSP treated splenocytes is a MPYS homodimer.

**Figure 3:**
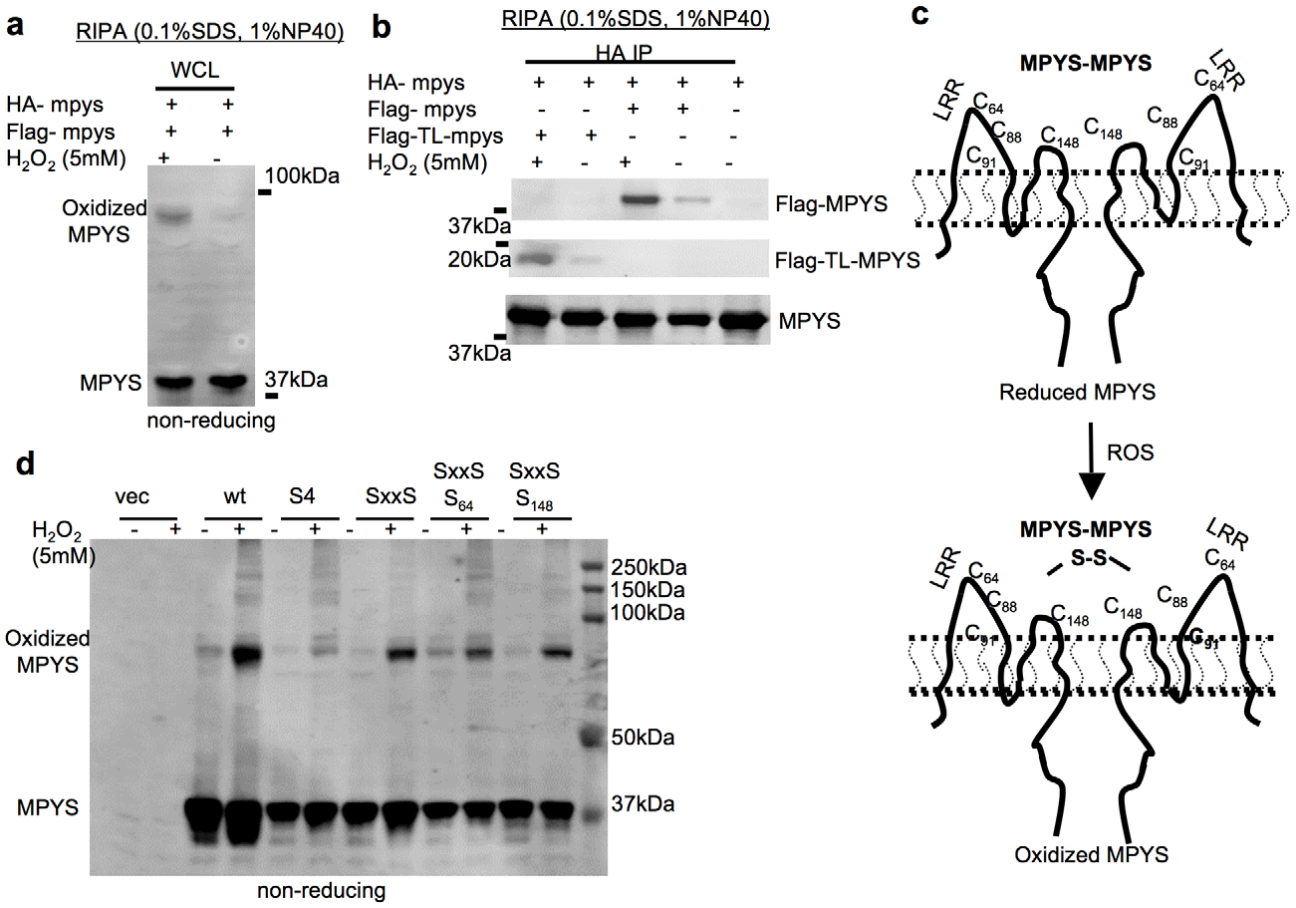
Homodimeric MPYS forms intermolecular disulfide bonds under oxidative stress. **a**. 293MT cells were co-transfected with indicated plasmids. After 48 hrs, the cells were treated with H_2_O_2_ (5 mM) at 37°C in PBS for 20 min. Cells were lysed in RIPA buffer, fractioned on a non-reducing SDS-PAGE gel and probed with anti-MPYS Ab as before. **b**. 293MT cells were co-transfected with indicated plasmids. After 48 hrs, the cells were treated as in (**a**) and lysed in RIPA buffer. HA IPs were done in the WCL and probed with indicated Abs. **c**. A cartoon illustrates the formation of oxidized MPYS under oxidative stress. Cysteines that may be involved in intermolecular disulfide bonds formation are listed. **d**. 293MT cells were co-transfected with indicated plasmids. Cells were treated as in (**a**) and lysed in RIPA buffer. WCL were run on a non-reducing gel. Oxidized MPYS is indicated.

Since in non-DSP treated cells this homodimer is sensitive to SDS (Figure 2d, lane 3), its “constitutive” dimerization is not mediated by intermolecular disulfide bonds. We conclude that MPYS exists in unperturbed cells primarily as non-disulfide bonded homodimer (Figure 3c, upper panel).

### MPYS becomes a disulfide-linked homodimer under conditions of oxidative stress

We wanted to know whether the non-disulfide-bonded SDS sensitive MPYS dimer becomes intermolecular disulfide bonded upon oxidation. Indeed we detected the similar ∼80 kDa SDS resistant MPYS in splenocytes after H_2_O_2_ treatment (Figure 2d, lane 4∼7). This oxidized MPYS is sensitive to the reducing SDS-PAGE conditions (Figure 2d, bottom panel) suggesting that it is mediated by disulfide bonds.

To determine whether the ∼80 kDa oxidized MPYS seen in cells subjected to oxidative stress is indeed a MPYS homodimer, we expressed both Flag-MPYS and HA-MPYS in the 293T cells. Cells were treated with H_2_O_2_ before lysis in the RIPA buffer containing 0.1% SDS. As expected, H_2_O_2_ treatment increased high-mass MPYS seen on non-reducing gels (Figure 3a). More importantly, anti-HA immunoprecipitates from H_2_O_2_ stimulated cells contained increased Flag-MPYS (Figure 3b) indicating that the oxidized MPYS is indeed disulfide bond linked MPYS homodimer (Figure 3c, lower panel).

### Cysteines in the ectodomain of MPYS are involved in the intermolecular disulfide bond formation

Interestingly, H_2_O_2_ treatment also increased the association of the Flag tagged tailless MPYS (Flag-TL-MPYS) with HA-MPYS (Figure 2b), which suggested that the Cysteines in the ectodomain of MPYS are involved in the formation of oxidized MPYS. Indeed, we found that a mutant MPYS (S4), in which all four cysteines in the ectodomain of MPYS were mutated to Ser (C64S, C88S, C91S and C148S) (Figure S3), had dramatically decrease oxidized MPYS formation after H_2_O_2_ treatment (Figure 3d). Comparing the H_2_O_2_ induced oxidized MPYS formation in S4, SxxS/S_64_ and SxxS/S_148_, it appeared that both the Cys64 and Cys148 are involved in the disulfide bond formation (Figure 3d).

### MPYS cysteines are critical for IFNβ stimulation

MPYS is an IFNβ stimulator [13]. Here, we showed that ROS induces intermolecular disulfide bonds formation in MPYS homodimer and inhibits its ability to activate IFNβ. We thus hypothesized that cysteines in MPYS are important for MPYS function. We mutate the critical cysteines involved in oxidized MPYS homodimer formation and examine their abilities to stimulate IFNβ promoter. In addition, we measured phosphorylation of IRF3 which lies downstream from MPYS in the IFNβ stimulation pathway [16].

**Figure 4:**
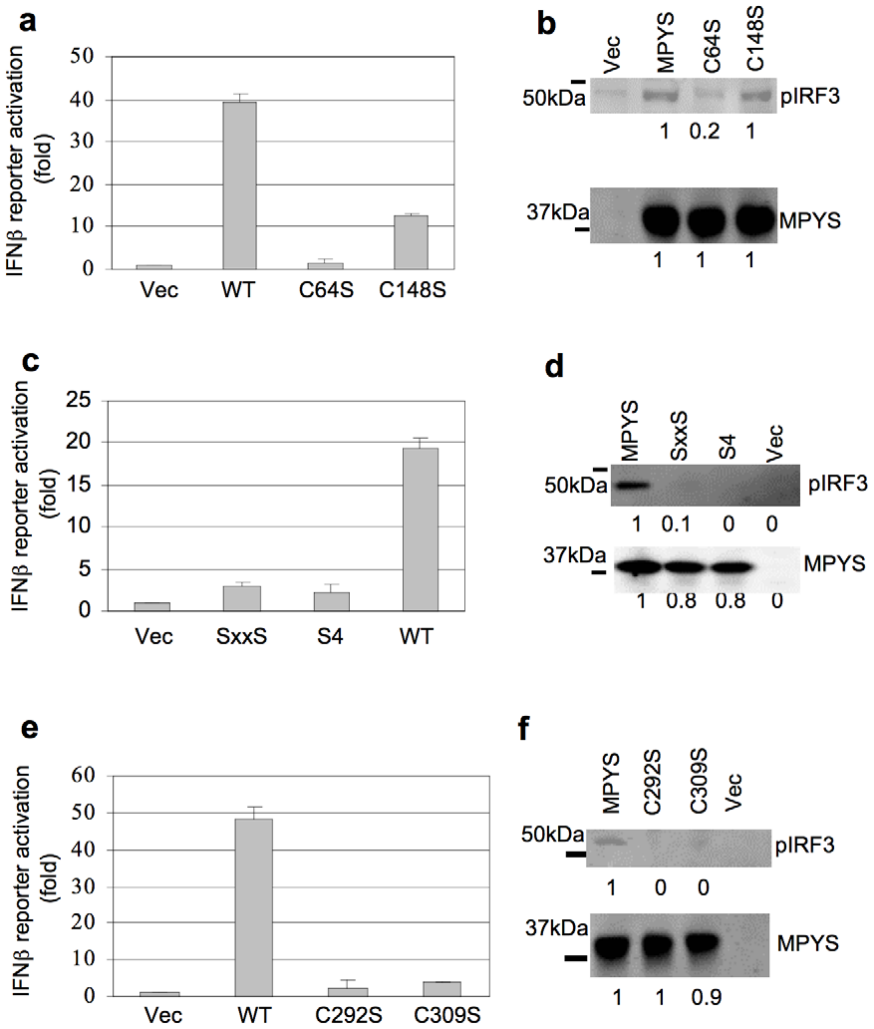
Cys-64, Cys-148, Cys-292, Cys-309 and C_88_xxC_91_ are required for IFNβ stimulation activity of MPYS. **a,c,e**. 293MT cells were transfected with indicated plasmid (100 ng) along with IFNβ-luciferase reporters. Luciferase activity was measured after 24 hrs as before. Error bars represent SD of a duplicate. **b,d,f**. 293MT cells were transfected with indicated plasmids. The blot was probed with anti-MPYS or p-IRF3 Ab (4D4G).

Mutation of these Cys to Ser revealed that the Cys64 is absolutely required for IFNβ reporter and IRF3 activation, while the Cys148 has moderate effect (Figure 4a, 4b). Mutation of the C_88_xxC_91_ to S_88_xxS_91_ or all four Cys (S4) eliminated MPYS ability to induce IRF3 phosphorylation and IFNβ production (Figure 4c, 4d).

To identify additional critical cysteines, we mutate the remaining 6 cysteines in MPYS. We found that the two cytoplasmic cysteines, Cys-292 and Cys-309, are also absolutely required for IRF3 activation and IFNβ stimulation (Figure 4e, 4f). We conclude that these 6 cysteines, Cys-64,-148,-292, -309 and C_88_xxC_91_, of MPYS are important for the induction of IFNβ (Figure S3).

## Discussion

In this report, we determined that MPYS, a potent stimulator IFNβ production, is a ROS sensor. We further found that the IFNβ stimulation by MPYS can be inhibited by high levels of ROS, and ROS stimulate MPYS oxidation as manifest by formation of intermolecular disulfide bonds within MPYS homodimers. We conclude that MPYS senses the Redox state of the cell and under conditions of high oxidative stress is “turned off”, presumably by oxidation.

ROS play an important role in regulation of innate immune responses and the strength of the ROS signals determines the outcome of such responses [1]. For example, low levels of H_2_O_2_ activate the p53 antioxidant response, whereas high levels trigger p53-dependent apoptosis [17]. The effect of ROS on MPYS function likely also depends on the levels of ROS. Rotenone treatment, which produces sustained cellular ROS, may cause irreversible modification on critical cysteines in MPYS thus inhibits the MPYS-mediated IFNβ response. Interestingly, NLRX1, a negative regulator of the anti-viral response [18], triggers strong ROS production (comparable to the level triggered by TNFα) [19]. Both NLRX1 and MPYS can be found in mitochondria [12], [19]. It is tempting to suggest that NLRX1 negatively regulates the IFNβ response by producing strong ROS, inhibiting MPYS activity.

MPYS contains 6 critical cysteines important for IFNβ stimulation. Among them, Cys-64, Cys-148 and C_88_xxC_91_ are targets of cellular ROS. The other two Cys are adjacent to Arg (Cys_292_-Arg_293_ and Cys_309_-Arg_310_). The positive charge of Arg293 or Arg310 may lower the p*K*a of Cys292 or Cys309 so that they may also be targets of ROS [20]. The question is, then, why these cysteines are important for IFNβ stimulation. Previous studies propose that MPYS/STING functions as an adaptor protein that recruits ser/thr kinase TBK1 [13], [21]. We find that TBK1 associates with both oxidized and reduced MPYS (Figure S4a). More importantly, both the C148S and C_88_xxC_91_ mutant have normal TBK1 association (Figure S4b). Considering the fact that the CxxC motifs are often found in the oxidoreductases and are essential for their catalysis of redox reactions [14], we hypothesize that MPYS may also have the oxidoreductase activity and TBK1 may be its substrate.

Previous over-expression studies have placed MPYS in the ER and mitochondrial outer membrane [12], [13], [16]. The mitochondrial outer membrane is physically and physiologically connected to the ER [22]. This physical link may facilitate Ca^2+^ [23] and, we suggest, ROS communication between ER and mitochondria. We found that the ER stress inducer, Brefeldin A, also generated oxidized MPYS (Figure S2a). Thus, MPYS may also act in ER stress sensing.

The mitochondrial intermembrane space (IMS) is connected to the cytosol by porins in the outer membrane of mitochondria which allow the diffusion of small ions like glutathione [24]. Thus the environment of IMS is less oxidizing than that of the ER lumen. Recently, a group of interacting mitochondrial proteins, including MPYS, VISA, NLRX1 and most recently Mitofusin 2, have been identified as key components of the innate intracellular viral sensing pathway [25] [18], [26]. In light of our current discovery that the MPYS is a Redox sensor, we suggest that the antiviral response also utilizes mitochondrial ROS as second messenger.

In conclusion, we have shown that the IFNβ stimulator, MPYS is a ROS sensor and its signaling function is regulated by ROS. Future studies need to be done to determine if MPYS is indeed an oxidoreductase and the *in vivo* biological significance of that activity.

## Materials and Methods

### Cell Culture

RAW264.7 [27], K46 B lymphoma cells [28] and the 293GT [29] cells were maintained in IMDM supplemented with 5% FBS as previously described [12]. The 293MT cells were sub-cultured from 293GT cells in DMEM (GIBCO, cat: 11965), 5% FBS (Biosource, 200p-500HI), sodium pyruvate (GIBCO 11360, 1 mM), HEPES buffer (GIBCO 15630-080, 10 mM) and 2-ME (50 µM) (Life Technologies, Gaithersburg MD). Sub-cultured 293GT cells that stimulate IFNβ production following MPYS overexpression were selected as 293MT cells.

RAW264.7 cells stably expressing the IFNβ-Luciferase construct were generated by co-transfecting RAW264.7 cells with pGL3-IFNβ-Luciferase (Promega) and p-Puromycin plasmids (Clonetch). The stable cell line was established by puromycin selection (4 µg/ml).

### Reporter Gene Assay

Cells were seeded in 24-well dishes (∼1×10^5^/ml) and transfected the following day using Effectene transfection reagent kit (Qiagen, 301427). Reporter assays were performed as previous described [30]. All experiments were repeated at least three times and results are representative. Error bars represent Standard deviation.

### Non-reducing SDS-PAGE

Cells were lysed in RIPA buffer (50 mM pH 7.4 Tris-Cl, 1% NP40, 0.25% sodium deoxycholate, 150 mm sodium chloride, 10 mM iodoacetamide, 0.1% SDS, 2 mM Na3VO4, 10 mM NaF, 0.4 mM EDTA, 1 mM PMSF, and 1 µg/ml each of aprotinin, α_1_-antitrypsin, and leupeptin). WCL equal to 40×10^5^ cells were mixed with 4× SDS loading buffer (100 mM Tris-Cl pH 6.8, 10% SDS, 20% glycerol and 0.2% bromphenol blue) and loaded directly on a NuPAGE 10% Bis-Tris Gel.

### Immunoprecipitation

Cells were lysed in 0.33% CHAPS buffer (150 mM NaCl, 10 mM Tris pH 7.5, 10 mM sodium pyrophosphate, 10 mM iodoacetamide, 2 mM Na_3_VO_4_, 10 mM NaF, 0.4 mM EDTA, 1 mM PMSF, and 1 µg/ml each of aprotinin, α_1_-antitrypsin, and leupeptin) at 4°C for 1 h. Cell lysates were centrifuged at 12,000 g at 4°C for 10 min. Immunoprecipitation was done in the lysates with indicated Ab-conjugated Sepharose beads.

### Measurement of intracellular ROS

Cells were washed and suspended in PBS, and then culture with H_2_DCFDA (5 µM) (Invitrogen, D399) for 20 min at 37°C. They were then washed in PBS and the fluorescence was measured by flow cytometry.

### RT-PCR

Human IFNβ (5′CAGCAATTTTCAGTGTCAGAAGC 3′ and 5′TCATCCTGTCCTTGAGGCAGT 3′) and HPRT1 (5′GGACAGGACTGAAAGACTTGCTCG 3′ and 5′TCCAACAAAGTCTGGCCTGTATCC 3′) were amplified using PrimeSTAR DNA polymerase (TaKaRa, R010A) under the following conditions: 94°C 30 sec, 30 cycles of 98°C 10 sec, 58°C 5 sec and 72°C 30 sec, then 72°C for 5 min.

### Listeria monocytogenes infection

RAW264.7 cells expressing the IFNβ-luc construct were infected with *Listeria monocytogenes* strain 10403S at multiplicity of five bacteria/cell. Cells were washed at 1 h after infection. Live bacteria were killed by addition of gentamicin. Whole cell lysates were prepared in RIPA buffer after infection. Luciferase activity was read with BD Monolight kit and Synergy reader.

## Supporting Information

Figure S1 **MPYS knockdown in RAW264.7 and K46 B cells. a, b.** WCL from K46 (**a**) or RAW264.7 (**b**) cells expressing either the luciferase control or MPYS knock-down constructs[12] were fractionated on a non-reducing SDS-PAGE, and blotted with anti-MPYS Ab. N.S.: non specific staining. **c.** RAW264.7-IFNβ-Luc cells expressing MPYS-knockdown (MPYS-KD) or control knock-down (Control) were infected with *Listeria monocytogenes* as in *Materials and Methods*. Luciferase activity was measured. P value was calculated by student T-test (one-tailed). **d**. RAW264.7-IFNβ-Luc cells were first treated with rotenone for 16hrs. Poly (I:C) (2.5µg/ml for 16hrs) was added into culture. Luciferase activity was measured as before.
(TIF)

Figure S2 **Endogenous MPYS becomes a disulfide-linked homodimer under oxidative stress.** **a**. RAW264.7 cells were treated with Brefeldin A (0.5µg/ml) for 20hr in culture. Cells were lysed in the RIPA buffer, fractionated using non-reducing SD-PAGE, and probed with anti-MPYS Ab. Oxidized MPYS is indicated. **b**. RAW264.7 cells were serum starved overnight. Cells were then harvested and lysed in RIPA buffer. The oxidized MPYS was detected as (**a**). **c.** 293MT cells were co-transfected with indicated plasmids. After 24 hrs, the cells were lysed in CHAPS buffer. Flag IP was performed. The blot was probed with anti-MPYS or anti-HA Ab.
(TIF)

Figure S3 **Alignment of MPYS from multiple species.** The human *mpys* cDNA described and used in this report was derived from a fetal liver library and found to differ by a single amino acid from that previously reported by [16] and [13] (indicated by arrow). In this sequence a G to A SNP (rs1131769) altered amino acid 232 from His (H) to Arg (R). The human population frequency data in the dbSNP database indicates that ∼80% of human are homozygous and ∼20% are heterozygous for this R232 allele. No European and only ∼2% of Japanese or Sub-Saharan African are homozygous for the previous reported H232 allele. Thus the R232 allele we studied here is the most relevant to the human population and referred as wild-type MPYS in this study. Cysteine residues important for the IFNβ stimulation were boxed.
(TIF)

Figure S4 **The C148S and CxxC mutants have normal TBK1 association.** **a**. Flag-TBK1 and MPYS were co-transfected into the 293MT cells. Flag proteins were precipitated. The immunoprecipitates were fractionated using non-reducing SDS-PAGE gels and blotted as indicated (left panel). WCL before or after the Flag IP were fractionated on a non reducing gel (right panel). Blots were probed with anti-MPYS Abs. **b**. Various HA tagged MPYS constructs were co-transfected with Flag-TBK1 into the 293MT cells. MPYS was immunoprecipitated by anti-HA MAb (16B12). Immunoprecipitates were fractionated and blots were probed with indicated Abs.
(TIF)
