# Supplementary figures and images for: Cellular Reactive Oxygen Species Inhibit MPYS Induction of IFNβ
Source: PLoS One. 2010 Dec 10;5(12):e15142. doi: 10.1371/journal.pone.0015142 (PMC3000824; doi:10.1371/journal.pone.0015142)

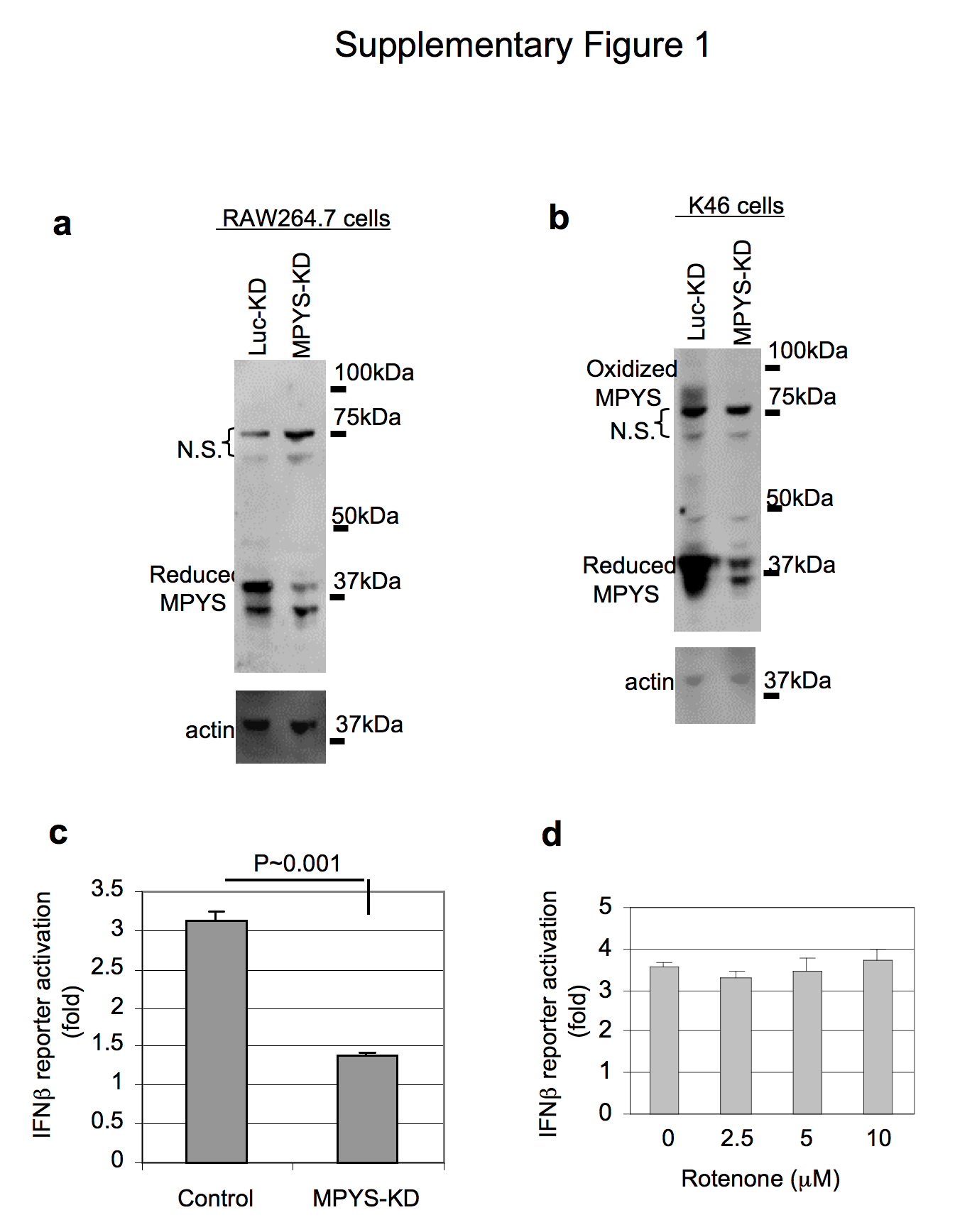

Supplement: Figure S1 — MPYS knockdown in RAW264.7 and K46 B cells. a, b. WCL from K46 (a) or RAW264.7 (b) cells expressing either the luciferase control or MPYS knock-down constructs[12] were fractionated on a non-reducing SDS-PAGE, and blotted with anti-MPYS Ab. N.S.: non specific staining. c. RAW264.7-IFNβ-Luc cells expressing MPYS-knockdown (MPYS-KD) or control knock-down (Control) were infected with Listeria monocytogenes as in Materials and Methods . Luciferase activity was measured. P value was calculated by student T-test (one-tailed). d. RAW264.7-IFNβ-Luc cells were first treated with rotenone for 16hrs. Poly (I:C) (2.5µg/ml for 16hrs) was added into culture. Luciferase activity was measured as before. (TIF) [file pone.0015142.s001.tif]

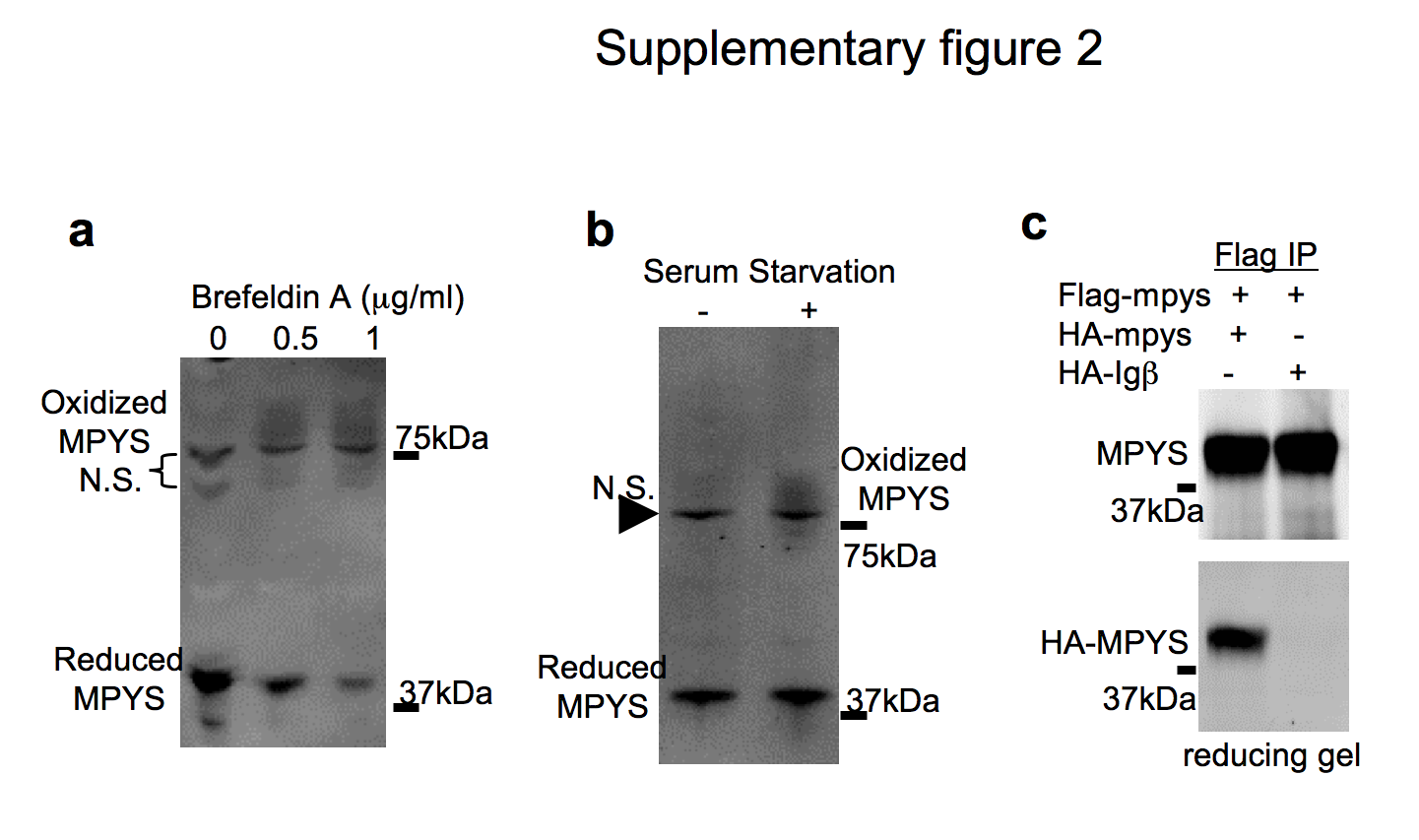

Supplement: Figure S2 — Endogenous MPYS becomes a disulfide-linked homodimer under oxidative stress. a. RAW264.7 cells were treated with Brefeldin A (0.5µg/ml) for 20hr in culture. Cells were lysed in the RIPA buffer, fractionated using non-reducing SD-PAGE, and probed with anti-MPYS Ab. Oxidized MPYS is indicated. b. RAW264.7 cells were serum starved overnight. Cells were then harvested and lysed in RIPA buffer. The oxidized MPYS was detected as (a). c. 293MT cells were co-transfected with indicated plasmids. After 24 hrs, the cells were lysed in CHAPS buffer. Flag IP was performed. The blot was probed with anti-MPYS or anti-HA Ab. (TIF) [file pone.0015142.s002.tif]

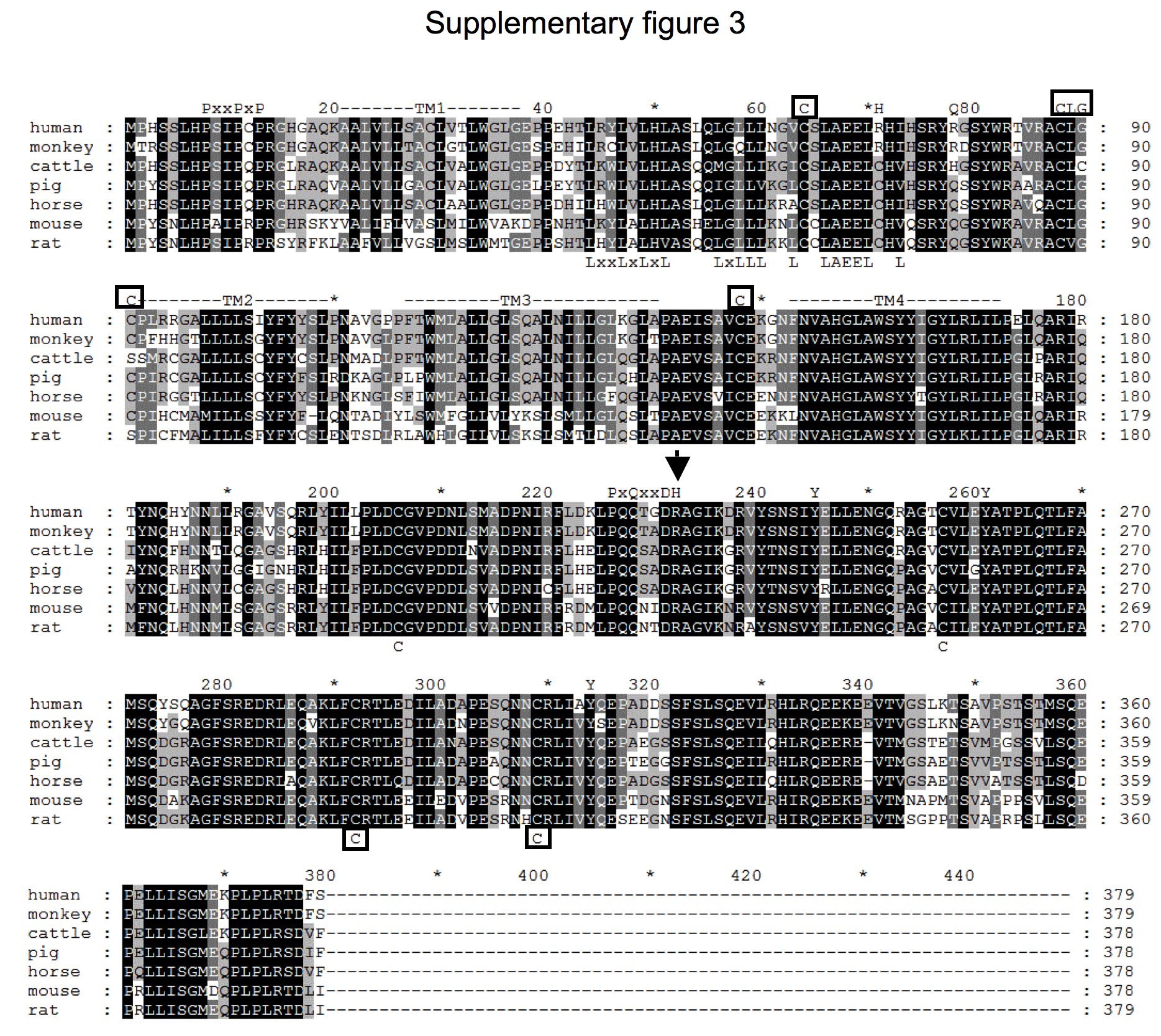

Supplement: Figure S3 — Alignment of MPYS from multiple species. The human mpys cDNA described and used in this report was derived from a fetal liver library and found to differ by a single amino acid from that previously reported by [16] and [13] (indicated by arrow). In this sequence a G to A SNP (rs1131769) altered amino acid 232 from His (H) to Arg (R). The human population frequency data in the dbSNP database indicates that ∼80% of human are homozygous and ∼20% are heterozygous for this R232 allele. No European and only ∼2% of Japanese or Sub-Saharan African are homozygous for the previous reported H232 allele. Thus the R232 allele we studied here is the most relevant to the human population and referred as wild-type MPYS in this study. Cysteine residues important for the IFNβ stimulation were boxed. (TIF) [file pone.0015142.s003.tif]

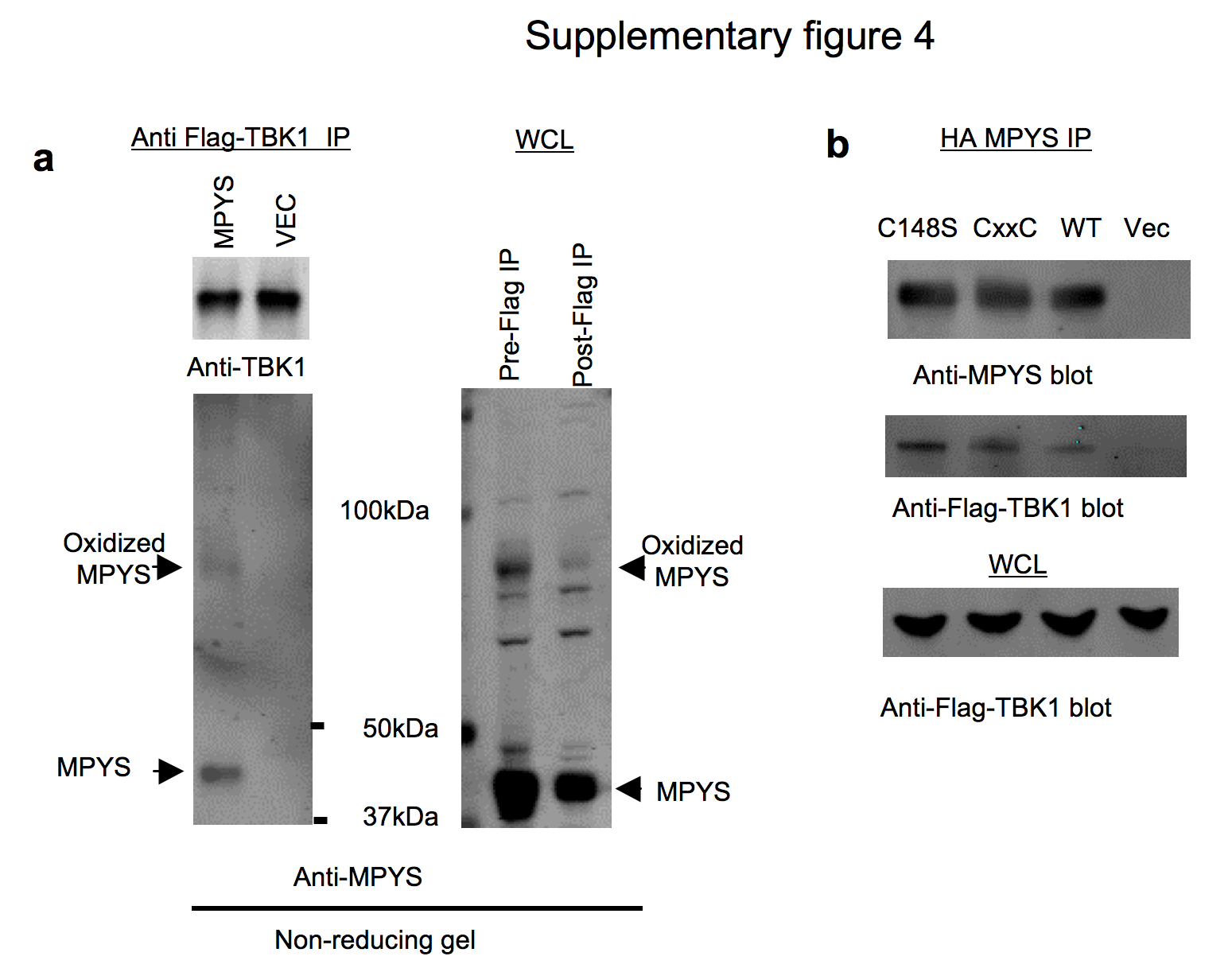

Supplement: Figure S4 — The C148S and CxxC mutants have normal TBK1 association. a. Flag-TBK1 and MPYS were co-transfected into the 293MT cells. Flag proteins were precipitated. The immunoprecipitates were fractionated using non-reducing SDS-PAGE gels and blotted as indicated (left panel). WCL before or after the Flag IP were fractionated on a non reducing gel (right panel). Blots were probed with anti-MPYS Abs. b. Various HA tagged MPYS constructs were co-transfected with Flag-TBK1 into the 293MT cells. MPYS was immunoprecipitated by anti-HA MAb (16B12). Immunoprecipitates were fractionated and blots were probed with indicated Abs. (TIF) [file pone.0015142.s004.tif]
